# Supplementary material for: Trends in Avoidable Hospitalizations for Heart Failure in Switzerland (1998–2018): A Cross-Sectional Analysis
Source: Healthcare (Basel). 2024 Dec 17;12(24):2547. doi: 10.3390/healthcare12242547 (PMC11675926; doi:10.3390/healthcare12242547)
Supplement: Supplementary file 1 [file healthcare-12-02547-s001.zip › healthcare-3297627-supplementary/Supplementary Tables.pdf]

SUPPLEMENTARY MATERIALS

Supplementary Table S1: Inclusion and exclusion criteria.

**Inclusion criteria (ICD-10 codes)**	**Exclusion criteria**
I11.0 Hypertensive heart disease with (congestive) heart failure<br>I13.0 Hypertensive heart and renal disease with (congestive) heart failure<br>I13.2 Hypertensive heart and renal disease with both (congestive) heart failure and renal failure<br>I50.0 Congestive heart failure<br>I50.1 Left ventricular failure<br>I50.9 Heart failure, unspecified	In-hospital deaths<br>Same-day hospitalisations (LOS=0)<br>Cases resulting from a transfer from another acute care institution.<br>Cases with cardiac procedure codes in any field: CHOP codes beginning with 35, 360, 361, 362, 363, 369 or 0066.<br>Cases with specified pregnancy, childbirth, and puerperium codes in any field: ICD-10 codes beginning with O

Source: *[13].*

Supplementary Table S2. Trends in the characteristics of potentially avoidable hospitalizations for heart failure, Switzerland, 1999-2018.

			Age group (%)						
**Year**	**N**	**Women (%)**	**[30-40[**	**[40-50[**	**[50-60[**	**[60-70[**	**[70-80[**	**[80-90[**	**[90+[**
1999	4,835	50.0	0.5	1.2	4.7	12.8	31.4	40.0	9.2
2000	5,421	51.1	0.5	1.4	5.2	13.2	32.3	37.9	9.4
2001	6,332	49.5	0.4	1.6	5.2	13.7	31.3	37.5	10.2
2002	6,864	50.3	0.3	1.1	5.0	11.8	31.7	39.9	10.1
2003	7,104	49.6	0.4	1.1	4.8	11.8	29.9	41.4	10.4
2004	7,812	50.1	0.2	1.2	4.4	11.6	30.0	41.5	11.0
2005	8,797	49.1	0.4	1.5	4.3	12.2	29.5	41.6	10.4
2006	9,810	48.9	0.4	1.2	4.7	11.7	28.7	42.1	11.1
2007	10,442	49.0	0.3	1.3	4.8	12.4	27.6	42.4	11.1
2008	10,770	50.0	0.2	1.2	4.2	11.3	28.8	42.7	11.4
2009	11,019	47.8	0.3	1.3	4.0	11.9	26.9	44.9	10.7
2010	11,516	49.0	0.3	1.4	4.5	11.5	27.3	42.9	11.9
2011	11,691	49.4	0.5	1.6	4.0	11.4	26.0	44.3	12.2
2012	11,875	48.3	0.4	1.4	4.0	11.0	26.4	42.9	13.8
2013	12,576	49.7	0.4	1.1	3.9	10.8	25.3	44.3	14.2
2014	13,138	49.0	0.4	1.2	3.8	10.6	25.5	43.5	14.9
2015	13,008	48.4	0.2	1.1	3.6	10.3	25.5	44.4	14.8
2016	14,043	49.1	0.3	0.9	3.4	10.1	24.3	44.7	16.1
2017	14,236	48.6	0.3	1.1	3.4	9.6	25.0	44.5	15.9
2018	14,711	48.6	0.3	1.0	3.8	9.2	25.2	44.2	16.3

N, number of admissions. Results are expressed as percentage relative to the number of admissions per year.

Supplementary Table S2 (continued). Trends in the characteristics of potentially avoidable hospitalizations for heart failure, Switzerland, 1999-2018.

Year	N	Swiss (%)	No insurance (%)	Emergency (%)	Decision (%) Patient's initiative	Ambulance	Doctor	Charlson's index >4 (%)
1999	4,835	87.5	4.7	60.7	13.9	12.4	61.7	16.9
2000	5,421	92.6	6.5	68.1	15.0	13.1	60.7	19.7
2001	6,332	90.2	5.0	71.3	14.1	13.6	63.9	20.9
2002	6,864	90.9	7.9	73.9	14.5	16.6	65.0	21.3
2003	7,104	90.4	4.7	76.9	19.3	16.9	61.5	22.4
2004	7,812	89.9	6.0	75.1	16.5	16.9	63.6	23.4
2005	8,797	89.6	6.4	77.3	17.1	17.4	62.0	28.3
2006	9,810	89.8	6.9	78.4	18.3	16.6	61.6	32.5
2007	10,442	89.7	4.6	76.7	18.2	17.8	62.6	35.4
2008	10,770	89.3	2.6	79.3	19.1	18.5	61.2	31.2
2009	11,019	88.8	2.1	80.7	20.1	20.8	57.7	38.6
2010	11,516	88.0	1.0	82.7	21.2	20.9	57.0	42.1
2011	11,691	87.9	0.9	83.7	22.9	21.5	55.0	33.2
2012	11,875	86.9	1.9	85.2	23.5	20.3	52.0	33.8
2013	12,576	86.9	0.8	87.1	24.6	20.9	50.1	35.4
2014	13,138	86.0	1.6	86.5	26.7	19.7	49.3	38.8
2015	13,008	86.1	0.7	88.9	28.1	21.4	46.8	41.4
2016	14,043	86.1	0.9	88.5	29.0	21.2	45.9	43.3
2017	14,236	85.5	0.9	88.4	30.2	22.1	43.8	45.1
2018	14,711	84.8	0.7	89.0	31.5	22.1	42.4	46.7

N, number of admissions. Results are expressed as percentage relative to the number of admissions per year.

Supplementary Table S3. factors associated with admission to the emergency ward among potentially avoidable hospitalisations for heart failure in Switzerland, 1999-2018.

	**OR (95% CI)**	**P-value**
Year	1.06 (1.06 - 1.06)	<0.001
Woman vs man	1.13 (1.10 - 1.16)	<0.001
Age category		
[20-30[	0.74 (0.55 - 1.01)	0.054
[30-40[	0.54 (0.46 - 0.65)	<0.001
[40-50[	0.78 (0.71 - 0.86)	<0.001
[50-60[	0.84 (0.79 - 0.89)	<0.001
[60-70[	1 (ref.)	
[70-80[	1.30 (1.25 - 1.35)	<0.001
[80-90[	1.66 (1.59 - 1.72)	<0.001
[90+[	1.89 (1.80 - 1.99)	<0.001
Non-Swiss vs. Swiss	1.07 (1.05 - 1.10)	<0.001
Region		
Léman	1 (ref.)	
Mittelland	1.03 (0.98 - 1.07)	0.225
Northwest	0.48 (0.46 - 0.50)	<0.001
Zurich	0.82 (0.78 - 0.85)	<0.001
Eastern	1.04 (1.00 - 1.09)	0.075
Central	1.63 (1.54 - 1.73)	<0.001
Tessin	0.79 (0.75 - 0.83)	<0.001
Charlson's index >4	1.15 (1.11 - 1.18)	<0.001
Decision for referral		
Patient's initiative	8.87 (8.45 - 9.30)	<0.001
Ambulance, police	6.55 (6.25 - 6.86)	<0.001
Doctor	1 (ref.)	
Other	0.95 (0.90 - 1.01)	0.097

Results are expressed as odds ratio (OR) and 95% confidence interval (95% CI).

Supplementary Table S4. Trends in the consequences of potentially avoidable hospitalizations for heart failure, Switzerland, 1999-2018.

Year	N	ICU (%)	LOS (days)	Discharged to Home	Med home	Other
1999	4,835	7.6	12 [8 - 18]	75.6	9.9	14.6
2000	5,421	8.9	12 [7 - 18]	76.3	9.5	14.2
2001	6,332	8.1	11 [7 - 17]	77.5	9.6	13.0
2002	6,864	10.0	11 [7 - 17]	73.6	9.6	16.8
2003	7,104	9.3	11 [7 - 17]	72.8	9.8	17.3
2004	7,812	9.2	11 [7 - 16]	71.2	10.2	18.6
2005	8,797	9.3	11 [7 - 16]	73.5	9.7	16.9
2006	9,810	7.9	11 [7 - 16]	73.0	9.6	17.4
2007	10,442	9.2	11 [7 - 16]	72.0	10.5	17.5
2008	10,770	8.4	11 [7 - 16]	73.1	11.2	15.8
2009	11,019	8.0	10 [7 - 15]	73.1	11.3	15.5
2010	11,516	8.2	10 [7 - 15]	73.6	11.5	14.9
2011	11,691	7.6	10 [7 - 15]	73.2	12.1	14.7
2012	11,875	8.5	10 [7 - 14]	71.9	14.1	14.0
2013	12,576	9.1	10 [7 - 14]	71.6	14.1	14.3
2014	13,138	9.0	10 [6 - 14]	70.9	14.1	15.1
2015	13,008	8.9	9 [6 - 14]	70.3	14.9	14.8
2016	14,043	9.1	9 [6 - 14]	69.8	14.7	15.4
2017	14,236	9.0	9 [6 - 13]	69.4	15.1	15.5
2018	14,711	8.6	8 [6 - 13]	70.0	15.1	14.9

N, number of admissions; ICU, intensive care; LOS; length of stay. Results are expressed as percentage relative to the number of admissions per year or as median and [interquartile range] for length of stay.

Supplementary Table S5. factors associated with admission to the intensive care unit among potentially avoidable hospitalisations for heart failure in Switzerland, 1999-2018.

	OR (95% CI)	P-value
Year	1 (1 - 1)	0.553
Woman vs man	0.90 (0.87 - 0.93)	<0.001
Age category		
[20-30[	2.29 (1.71 - 3.08)	<0.001
[30-40[	1.59 (1.31 - 1.94)	<0.001
[40-50[	1.53 (1.37 - 1.70)	<0.001
[50-60[	1.16 (1.08 - 1.24)	<0.001
[60-70[	1 (ref.)	
[70-80[	0.65 (0.62 - 0.68)	<0.001
[80-90[	0.33 (0.32 - 0.35)	<0.001
[90+[	0.12 (0.11 - 0.13)	<0.001
Non-Swiss vs. Swiss	1.00 (0.97 - 1.02)	0.725
Region		
Léman	1 (ref.)	
Mittelland	0.51 (0.49 - 0.54)	<0.001
Northwest	0.55 (0.52 - 0.58)	<0.001
Zurich	0.52 (0.50 - 0.55)	<0.001
Eastern	0.42 (0.40 - 0.45)	<0.001
Central	0.91 (0.85 - 0.96)	0.002
Tessin	1.04 (0.98 - 1.10)	0.238
Charlson's index >4	1.24 (1.20 - 1.28)	<0.001
Decision for referral		
Patient's initiative	1.17 (1.12 - 1.21)	<0.001
Ambulance, police	2.06 (1.98 - 2.14)	<0.001
Doctor	1 (ref.)	
Other	0.80 (0.72 - 0.88)	<0.001
Via emergency ward vs. other	1.98 (1.88 - 2.09)	<0.001

Results are expressed as odds ratio (OR) and 95% confidence interval (95% CI).
