# Supplementary figures and images for: Trends in Avoidable Hospitalizations for Heart Failure in Switzerland (1998–2018): A Cross-Sectional Analysis
Source: Healthcare (Basel). 2024 Dec 17;12(24):2547. doi: 10.3390/healthcare12242547 (PMC11675926; doi:10.3390/healthcare12242547)

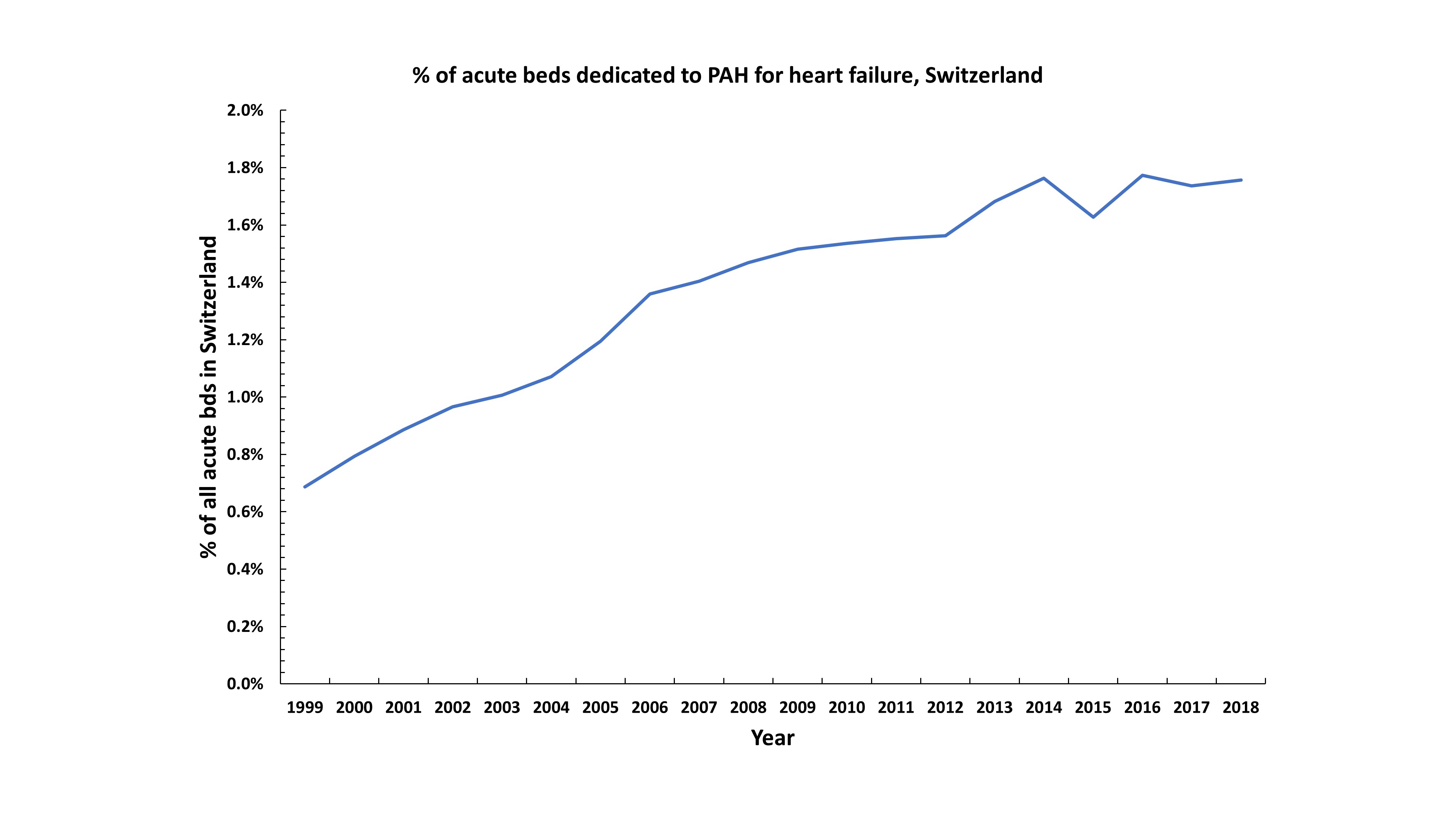

Supplement: Supplementary file 1 [file healthcare-12-02547-s001.zip › healthcare-3297627-supplementary/Supplementary Figure.jpg]
